# Effects of Basketball and Baduanjin Exercise Interventions on Problematic Smartphone Use and Mental Health among College Students: A Randomized Controlled Trial

**DOI:** 10.1155/2021/8880716

**Published:** 2021-01-28

**Authors:** Tao Xiao, Can Jiao, Jie Yao, Lin Yang, Yanjie Zhang, Shijie Liu, Igor Grabovac, Qian Yu, Zhaowei Kong, Jane Jie Yu, Jieting Zhang

**Affiliations:** ^1^College of Mathematics and Statistics, Shenzhen University, Shenzhen 518060, China; ^2^Center for Lifestyle and Mental Health, School of Psychology, Shenzhen University, Shenzhen 518060, China; ^3^School of Humanities and Social Sciences, Harbin Institute of Technology (Shenzhen), Shenzhen 518055, China; ^4^Department of Cancer Epidemiology and Prevention Research, Cancer Care Alberta, Alberta Health Services, Calgary, AB, Canada; ^5^Departments of Oncology and Community Health Sciences, Cumming School of Medicine, University of Calgary, Calgary, AB, Canada; ^6^Exercise and Mental Health Laboratory, Shenzhen University, Shenzhen 518060, China; ^7^Health and Exercise Science Laboratory, Institute of Sports Science, Seoul National University, Seoul, Republic of Korea; ^8^School of Physical Education, Jianghan University, Wuhan 430056, China; ^9^Department of Social and Preventive Medicine, Centre for Public Health, Medical University of Vienna, Vienna, Austria; ^10^Faculty of Education, University of Macau, Macao, Zhuhai, China; ^11^Department of Sport and Exercise Science, College of Education, Zhejiang University, Hangzhou 310058, China; ^12^School of Psychology, Shenzhen University, Shenzhen 518060, China

## Abstract

Problematic smartphone use (PSU) has become a prevalent issue worldwide. Previous studies suggest that physical exercising may effectively reduce smartphone users' addiction levels. Comparisons and further evaluations on the long-term effects of different types of exercise-based interventions on treating PSU remain to be investigated. *Objective*. We investigated if group-based basketball and Baduanjin exercise (a type of Qigong) would reduce PSU and improve the mental health of college students and whether such effects would be sustained. A twelve-week experiment was conducted, where 96 eligible Chinese college students with PSU were randomly assigned to two intervention arms (i.e., basketball and Baduanjin exercises) and a control arm. Outcome measures, including PSU (measured by the Mobile Phone Addiction Index in Chinese (MPAI)) and mental health indices for anxiety (measured by Self-Rating Anxiety Scale (SRAS)), loneliness (measured by the short-form of the UCLA Loneliness Scale (UCLA-LS)), inadequacy (measured by the revised Janis and Field's Feelings of Inadequacy Scale (FIS)), and stress (measured by the Chinese version of Perceived Stress Scale (CPSS)) were collected at the baseline, the end of week 12, and the two-month follow-up. A Generalized Estimating Equations (GEE) model for longitudinal data was utilized in analyses. *Results*. Both exercise interventions demonstrated significant effects on decreasing PSU (basketball: *p* < 0.01; Baduanjin: *p* < 0.01), feelings of anxiety (basketball: *p* < 0.01; Baduanjin: *p*=0.04), loneliness (basketball: *p* < 0.01; Baduanjin: *p* < 0.01), inadequacy (basketball: *p* < 0.01; Baduanjin: *p* < 0.01), and perceived stress (basketball: *p* < 0.01; Baduanjin: *p*=0.04), at the end of interventions. At two months after interventions, both exercise interventions demonstrated significant effects on decreasing PSU (basketball: *p* < 0.05; Baduanjin: *p* < 0.05), feelings of anxiety (basketball: *p* < 0.01; Baduanjin: *p*=0.03), loneliness (basketball: *p* < 0.01; Baduanjin: *p* < 0.01), and inadequacy (basketball: *p* < 0.01; Baduanjin: *p*=0.01), but not for feeling of stress. Furthermore, group-based basketball demonstrated larger improvements for all these significant results on reducing PSU and meanwhile improving their related mental health parameters among college students.

## 1. Introduction

With the development of smartphone technology in recent decades, the number of smartphone users has grown dramatically throughout the world. However, the availability of smartphones also brought about the increasingly prevalent issue of problematic smartphone use (PSU), which displays common signs related to addiction, such as craving, lack of control, and withdrawal symptoms [1]. Recent studies reported that individuals with PSU encountered various physical, psychological, and social problems that could do great harm to their lives [2–4], particularly for vulnerable youths (including college students) who account for a significant proportion of the affected population worldwide [5, 6]. For example, 20% of teenagers in Spain, up to 36% in the UK [7], and 21% in China [8] were found to have PSU, while the prevalence rates were reported with 62.3% and 64.9% for female and male college students, respectively [9, 10].

Tackling PSU is still under investigation; however, given the commonalities with other addictive behaviors, it may benefit from therapeutic approaches focused primarily on prescription medication and cognitive behavioral therapy. Alternatively, exercise interventions are also gaining popularity given findings reporting the effectiveness in treating individuals with substance abuse [11, 12]. Physical exercise is a safe and feasible treatment alternative that is likely to reduce PSU and improve comorbid mental health problems. As frequent smartphone use is associated with less physical activity, time spent exercising can both reduce the time of phone use and meanwhile induce similar reward-based effects as phones. Furthermore, as exercise is often a social activity, promoting more social encounters, it may also contribute to overall mental wellness. According to a recent meta-analysis of randomized controlled trials [7] on exercise interventions including typical aerobic exercises such as running, bicycling, basketball, badminton, football, tennis, and dancing, as well as aerobic exercises containing mindful movements such as Baduanjin and Taichi, doing physical exercising was found with evidence to effectively reduce the smartphone users' addiction levels, and prolonged engagement in exercise was associated with fewer withdrawal and mood-related symptoms.

These findings are promising, but with further demand to investigate the comprehensive healing effects of exercise interventions on PSU. First, people with PSU were often found to be more socially anxious and lonelier and have lower self-esteem and higher stress levels [13, 14]; hence, understanding changes in the mental wellness, alongside changes of absolute levels of addictive smartphone use [7, 15], can add more insights. Second, research is needed to confirm the long-term effects of physical activities on reducing PSU, as most of the existing studies did not have postintervention follow-up assessments. Third, it is worthy of further investigation on whether and how different types of exercise interventions for mental health (e.g., [16–26, 27]) can address these psychological problems and maintain the influence, in order to assist the development of more tailored programs with personalized feasibility considered to better help people in different mental health states. In particular, evaluating and comparing oriental exercise interventions containing mindful movements (e.g., Tai Chi, Yoga, or other types of Qigong exercises) and other widely played sports which originated in the West (e.g., basketball) with regard to treating PSU can be very valuable in consideration of their mechanism differences.

**Figure 1:**
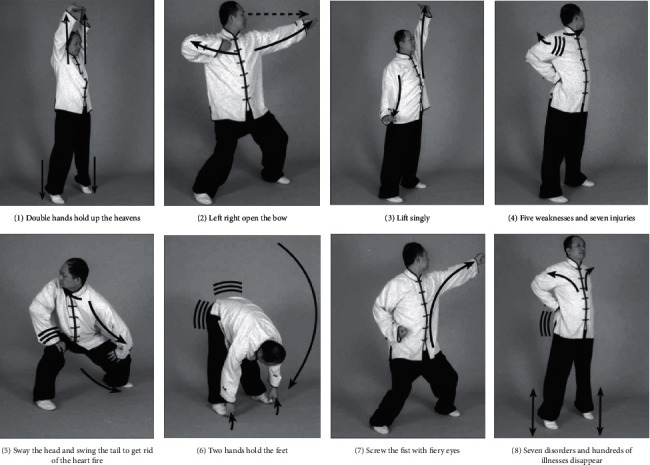
Snapshots of eight components of movements of Baduanjin.

Baduanjin (or Eight Pieces of Brocade), created more than one thousand years ago during the Chinese Southern Song dynasty, is a Qigong exercise containing eight components of mindful movements and is easy to learn. These eight pieces are, namely, (1) Double Hands Hold up the Heavens, (2) Left Right Open the Bow, (3) Lift Singly, (4) Five Weaknesses and Seven Injuries, (5) Sway the Head and Swing the Tail to get rid of the Heart Fire, (6) Two Hands Hold the Feet, (7) Screw the Fist with Fiery Eyes, and (8) Seven Disorders and Hundreds of Illnesses Disappear. Snapshots of these eight movements (postures may vary stylewise) illustrated by Yang are provided in Figure 1 [28]. On the other hand, basketball, invented relatively much recently (1891) by Canadian-American gym teacher James Naismith in the United States, has evolved to become one of the world's most popular sports [29]. As a valuable comparison of mental health healing effects by different types of exercise interventions, the present study aimed to examine the impacts of these two typical exercises on reducing PSU of college students, who are at a higher risk for developing lifelong unhealthy habits. Both 12-week intervention effects and follow-up assessments were to be quantified, in order to assess the strength and sustainability of such effects. Another objective was to determine psychological changes measured by indicators of anxiety, loneliness, inadequacy, and stress, as a result of exercise interventions. Aligning such mental health aspects with smartphone use, this study sought to confirm the overall positive influences of exercising on youth with PSU, as well as exploring psychological pathways to reduce their addictive behaviors.

## 2. Methods

### 2.1. Participants

A total of 762 students were recruited from a comprehensive university in central China via advertisement and social media, China, to complete the Mobile Phone Addiction Index (MPAI), which was used to identify PSU [30]. Those with MPAI scores above 40 accounted for 22.2% of all respondents and were considered as potential candidates for the interventions. To be finally included, these potential candidates were evaluated against the inclusion criteria. Firstly, given the exercise training requirement, they must be able to independently ambulate without any assisting device. Secondly, they had no major disease (cardiovascular disease, respiratory illness, and musculoskeletal disorder) that can affect them to participate in exercise training. Thirdly, they were not diagnosed with any psychiatric (e.g., depression, anxiety, bipolar disorder, obsessive-compulsive disorder, eating disorder, and posttraumatic stress disorder) and attention disorders. Fourth, they did not attend any structured exercise training in the past three months, including no basketball and mindful exercise training experience prior to this study. Eventually, 100 qualified candidates were randomly assigned to three groups using the Excel RAND function: basketball (*N* = 33), Baduanjin (*N* = 33), and the control group (*N* = 34). However, due to scheduling conflict (*N* = 2), injury caused by other things (*N* = 1), and studying abroad (*N* = 1), only 96 participants were involved in the whole intervention period and therefore Per Protocol analysis was used.

### 2.2. Measures

The main outcomes of interest in this study were problematic smartphone use and related psychological indicators including anxiety, loneliness, inadequacy, and stress, which were measured with widely used instruments. The questionnaire is divided into 6 parts, which are basic information (age, gender, profession, whether or not they are the only children, etc.), mobile phone addiction index, self-rating anxiety scale, loneliness scale, feelings of inadequacy scale, and perceived stress scale, totaling about 25 minutes to occupy your time.

#### 2.2.1. Primary Outcomes (Problematic Smartphone Use (PSU))

PSU was measured by the MPAI in Chinese [14,^30^]), which includes 17 items addressing four aspects of addictive behavior, that is, inability to control cravings, anxiety and feeling lost, withdrawal and escape, and productivity loss. All questions are rated on a 5-point scale, and a higher total score indicates a more severe level of problematic use.

#### 2.2.2. Secondary Outcomes

Anxiety was measured by the Self-Rating Anxiety Scale or SRAS [31], which is a norm-referenced scale commonly used to identify the presence of anxiety. According to the norms in China, scores above 50 indicate anxiety, with 50–59 as minor anxiety, 60–69 as moderate anxiety, and 70 and above as severe anxiety [32]. Loneliness was measured using the short-form of the UCLA Loneliness Scale (UCLA-LS), which includes 8 items and has been validated among Chinese college students [33]. A higher total score indicates a higher level of perceived loneliness. Inadequacy refers to low self-esteem and absence of self-confidence in various social contexts and was measured by the revised Janis and Field's Feelings of Inadequacy Scale (FIS) [34]. It should be noted that higher scores on this scale refer to lower levels of inadequacy. As one of the most frequently used tools to quickly measure perceived stress, the original Perceived Stress Scale (PSS-14), as well as its Chinese version (CPSS), proved to have satisfactory psychometric properties [35]. Therefore, respondents were asked to complete the CPSS and report their stress levels regarding different life aspects.

### 2.3. Procedure

**Figure 2:**
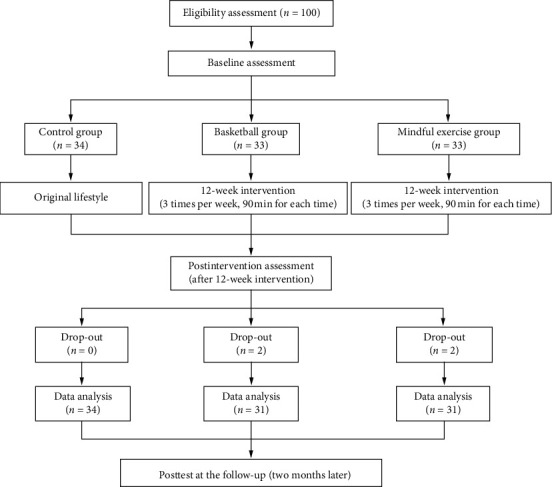
Flowchart displaying the process of randomization, intervention, and follow-up assessment.

This study was a twelve-week randomized controlled study with two experimental conditions (i.e., basketball exercise and Baduanjin exercise). Participants in the experimental arms were engaged in either basketball or Baduanjin exercise sessions for twelve weeks, which lasted 90 minutes each time and occurred three times each week. In comparison, the control group members maintained their routines and received no intervention. As mentioned earlier, four participants did not complete the interventions due to different reasons and therefore were not able to receive postintervention assessment (i.e., two drop-outs from each of the two experimental arms in Figure 2). The other 96 study participants were assessed at the baseline, immediately after the intervention (12 weeks later), and at the follow-up (two months later). Prior to this intervention, all eligible participants had signed the consent forms approved by the ethical committee of the university.

Participants in the basketball group received basketball training taught by one professional basketball coach from the university. They participated in exercise three times per week for 12 weeks. Each session included 10 minutes of warm-up, 70 -minutes of basketball exercise (moderate intensity: 120–150 b/min), and 10 minutes of cooldown. The training program consisted of basic movements, basketball skills (lay-ups, dribbling, and shooting), and games. Basketball was played as 3 versus 3 (or 2 versus 2) on half court. Given that the beneficial effects of Baduanjin on mental health have been well documented [26, 36, 37], its form was arranged in the other experimental group. A certified instructor from the university taught and supervised the participants, who participated in exercises three times per week for 12 weeks. Each session included 10 minutes of warm-up, 70 minutes of Baduanjin practice, and 10 minutes of cooldown. The whole set of Baduanjin included eight postures, consisting of the starting and ending postures.

### 2.4. Statistical Analysis

For each outcome of interest, a population average model using generalized estimating equations or GEE [38] was built with the Stata16 software [39], to assess the intervention effects of the two exercise interventions comparing with control over time, while adjusting for potential confounders. Correlations among the three repeated measures of each outcome variable taken within each participant were assumed to be exchangeable, and parameter estimates and their robust standard errors were obtained with the Stata command *xtgee* for the GEE approach, for which the distribution assumption of the dependent variable is not required [40]. Main and interaction effect terms for both the repeated measure time factor and the group factor were included in the GEE model for each outcome at first, and then we assessed the significance of the time-arm interaction for each outcome. If the time-arm interaction effects for an outcome were significant, we report estimated coefficients and their *p*-values for both the main effects and the interaction effects for this outcome. Distribution inequality among the three RCT arms was detected for each baseline variable, and baseline variables that turned out to be significant would be included as main effect terms in all GEE models to control for their confounding effects. Statistical significance was set at type I error level of 0.05. For each of all the five outcome variables (i.e., MPAI and four mental health indices), means and standard deviation ranges of raw values at different time points in each of the three RCT groups are illustrated in interaction plots. Also given in each interaction plot are “×” symbols at different postintervention time points, which indicate the significance of the corresponding “time × intervention” interaction effect for that time point by the Walt test of the GEE model, representing a significant intervention effect across the time span from baseline to that postintervention time point as compared to the control group. That is to say, each “time × intervention” interaction term measures the difference between “the change of outcome from the baseline to the corresponding time in the corresponding intervention group” and “the change of outcome from the baseline to the corresponding time in the control group,” and thus a significant interaction term indicates a significant change of outcome induced by the intervention during this time span (from the baseline to the corresponding time) as compared to the control group.

### 2.5. Ethics

The study procedures were carried out in accordance with the Declaration of Helsinki. The Institutional Review Board of the university approved the study. All subjects were informed about the study and all provided informed consent.

## 3. Results

### 3.1. Demographic Information

Distribution inequality among the three randomized arms was tested for each of baseline variables, that is, age, gender, grade/college level, major, family, class role, dating, monthly mobile phone fee, daily mobile phone use, purpose of mobile phone use, main social use of mobile phone, and awareness of harm of mobile radiation. Only major turned out to have group difference (*p* < 0.05), and thus major was included as a main effect term in all GEE models to control for its confounding effect. Detailed demographic information is presented in Table 1.

### 3.2. Respondent Characteristics across Groups

For each of the three groups, Table 2 displays respondents' average scores on smartphone use and the four psychological measures at the three time points. All three groups started at similar levels of PSU, anxiety, stress, inadequacy, and loneliness, yet there were improvements across all indicators at the end of the interventions in the two experimental groups. For example, the MPAI scores of Baduanjin participants dropped from 51.13 to 42.74 after 12 weeks, suggesting a moderate reduction in their PSU levels. In comparison, the control group only witnessed small fluctuations in scores of all five measures from the baseline to week 12. Furthermore, when comparing baseline against the 2-month follow-up scores, positive changes in smartphone use and psychological indicators were still evident among the Baduanjin and basketball groups.

### 3.3. Effects of Exercise Interventions

**Figure 3:**
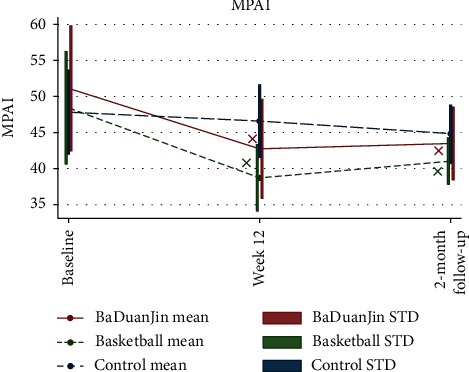
Interaction plots for comparisons of intervention effects on mobile phone addiction index.

**Figure 4:**
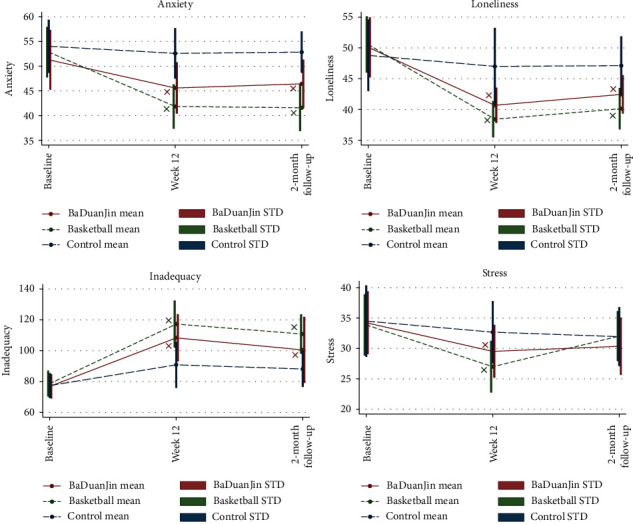
Interaction plots for comparisons of intervention effects on mental health indices.

The regression coefficient estimation results by the GEE model for all of the five outcomes are organized in Table 3. Since this is a randomized controlled trial, the group means can be assumed to be equal at baseline and the test of the intervention effect is subsumed within the test of the “time × intervention” interaction. As can be seen in the “Interaction effect” section of Table 3, significant effects for all outcomes right after intervention were observed for both Baduanjin and basketball groups. These significant effects stayed for at least two months beyond the intervention period, with the only exception of stress. These trends with corresponding significance indicators are also illustrated in the interaction plots of all five outcome variables in Figures 3 and 4. Quantitative interpretations of significant intervention effects for the five outcome variables in Table 3 are given next, categorized by short-term or long-term effects with regard to the two postintervention time points, respectively.

#### 3.3.1. Short-Term Effects of Exercise Interventions

PSU: both Baduanjin and basketball contributed to significantly larger reductions of PSU level as compared to the control group at the end of the intervention period; see Table 3 and Figure 3. Specifically, the MPAI scores dropped 8.47 more among basketball participants (95% CI −12.34 to −4.61, *p* < 0.01) and 7.15 more among Baduanjin participants (95% CI −10.48 to −3.82, *p* < 0.01) than those receiving no intervention, suggesting a strong short-term effect of both exercising interventions on reducing PSU; see Table 3 and Figure 3.

Psychological outcomes: all four mental health indicators were found to be positively influenced by both Baduanjin and basketball interventions significantly; see Table 3 and Figure 4. Participants' levels of anxiety, loneliness, inadequacy, and perceived stress dropped noticeably from baseline to week 12 as a result of exercising, comparing to the control group. Specifically, from baseline to week 12, those who played basketball regularly has a reduction of 9.56 (95% CI −12.87 to −6.25, *p* < 0.01) on anxiety score (SRAS), a reduction of 10.33 (95% CI −13.79 to −6.88, *p* < 0.01) on loneliness score (UCLA-LS), an increase of 25.14 (95% CI 17.43 to 32.85, *p* < 0.01) on inadequacy score (FIS), and a reduction of 5.05 (95% CI −7.52 to −2.57, *p* < 0.01) on stress score (UCLA-LS), more than that among the control group. Similarly but to less extent, from baseline to week 12, Baduanjin participants witnessed drops in these four mental health indices as well: those who played Baduanjin regularly have a reduction of 4.27 (95% CI −8.23 to −0.31, *p*=0.04) on anxiety score (SRAS), a reduction of 7.63 (95% CI −11.29 to −3.96, *p* < 0.01) on loneliness score (UCLA-LS), an increase of 18.01 (95% CI 9.46 to 26.56, *p* < 0.01) on inadequacy score (FIS), and a reduction of 2.89 (95% CI −5.61 to −0.16, *p* < 0.01) on stress score (UCLA-LS), more than that among the control group. Overall, both basketball and Baduanjin proved to be most beneficial in alleviating loneliness and feelings of inadequacy, as indicated by the strongest changes in the two measures in Table 3 (*p* < 0.01) and Figure 4.

#### 3.3.2. Long-Term Effects of Exercising Interventions

PSU: even after the interventions were over, participants still benefited from both exercise interventions, whose MPAI scores two months later remained significantly lower than their baseline scores. As Table 3 and Figure 3 display, MPAI scores of basketball and Baduanjin participants were, respectively, reduced 4.42 (95% CI −7.86 to −0.98, *p* < 0.05) and 4.67 (95% CI −8.23 to −1.12, *p* < 0.05) more than those in the control group, proving the lasting effect of exercising in the long run. Compared with Week 12 scores, MPAI scores of Baduanjin participants were somewhat more stable than those of basketball participants at the two-month follow-up, suggesting that the influence of Baduanjin on PSU might be more enduring.

Psychological outcomes: significant effects of exercise interventions on participants' mental health were carried over two months later after the end of interventions, with the only exception of perceived stress. Loneliness and feelings of inadequacy were still improved to the greatest extent in both exercising groups. Specifically, from baseline to 2-month follow-up after intervention, those who played basketball regularly has a reduction of 10.08 (95% CI −13.02 to −7.14, *p* < 0.01) on anxiety score (SRAS), a reduction of 8.74 (95% CI −12.05 to −5.44, *p* < 0.01) on loneliness score (UCLA-LS), and an increase of 21.36 (95% CI 13.64 to 29.09, *p* < 0.01) on inadequacy score (FIS), more than that among the control group. Similarly but to less extent, from baseline to week 12, Baduanjin participants witnessed drops in these four mental health indices as well: those who played Baduanjin regularly has a reduction of 3.69 (95% CI −6.92 to −0.46, *p*=0.03) on anxiety score (SRAS), a reduction of 5.97 (95% CI −9.17 to −2.76, *p* < 0.01) on loneliness score (UCLA-LS), an increase of 12.88 (95% CI 3.73 to 22.03, *p* < 0.01) on inadequacy score (FIS), more than that among the control group. On the other hand, no significance was found regarding the improvements in perceived stress at the two-month follow-up as compared to baseline for both exercising groups (*p*=0.30 for Baduanjin group and *p*=0.56 for the basketball group), when the reduction in stress levels from the baseline was no longer stronger than that in the control group.

## 4. Discussion

As demonstrated by previous experimental research, exercise engagement for at least 12 weeks was effective in reducing addictive smartphone use and withdrawal symptoms [7]. What remained unclear was the lasting effect of exercise beyond the end of intervention, as well as differences in the effectiveness of different types of exercises, especially those between traditional oriental exercises containing mindful movements and team sports such as basketball.

The results of this study showed that PSU of the participants in both intervention groups were reduced after the intervention and such beneficial effects were sustained for two months. These findings confirm the importance of exercising as an alternative approach for alleviating PSU, which also contributed to the improvement of mental health outcomes, particularly on anxiety, inadequacy, and loneliness. These findings also accord with previous researches that attendance of both team sports (e.g., [41]) and exercises containing mindful movements (e.g., [16, 20, 21, 23, 25]) are significantly associated with mental health.

The beneficial effects varied between the two intervention types based on our data, which is worthy of some notes and further investigations. Group-based basketball intervention was observed to have a greater effect on reducing PSU and improving mental health outcomes. Part of the reason might be that social activities involved in the group-based basketball help relieving mental health burden than exercise such as Baduanjin without interacting with others. In addition, group-based basketball intervention is generally more vigorous and involved with more competition as compared to Baduanjin, and hence group-based basketball might be associated with a higher level of dopamine increase to combat with addiction of smartphone use [42]. However, Baduanjin seems to yield more enduring effect on lowering PSU, with potential reason that the training of mindful movements typically in accords with rhythmic deep breaths could help improve one's discipline and activity endurance for a longer run [43].

Other than the effects of interventions, the feasibility is also an important factor for exercise intervention promotion among people with PSU. Comparing to basketball, Baduanjin is a convenient (practice time can be as less as 10 to 20 minutes) and noninvasive exercise that attracts many peoples' attention in real life [28]; recommending Baduanjin exercise to people with addictive smartphone use appears to be more feasible than basketball. In particular, while practicing Baduanjin exercise, these participants may improve their confidence and pleasant moods to make greater efforts. As it positively cultivates the ability of self-control in their moods and helps them enjoy this exercise mode, their mental health issues such as anxiety and loneliness will possibly disappear. Collectively, Baduanjin exercise seems to be an available approach that could be effectively used for alleviating mood disorders in people with PSU.

Although both Baduanjin and basketball interventions in our study are effective in reducing smartphone use of college students and improving their mental health, the main limitations of this study are stated as follows. Firstly, a limited sample was obtained from only one regional university in China. Therefore, the results may not be generalizable before we replicate the study among other Chinese students or student populations in other countries, especially without culture-related confounders in consideration. We encourage researchers to examine the effects of culture-specific physical exercise on population of different regions globally to adjust for such a confounder effect [44]. Secondly, follow-up positive effects were observed, but two months may be too short to determine and compare the long-term effect of Baduanjin and basketball. Relapse seems to be common for PSU and hence research designs with longer follow-up time are in demand and better equipped with state-of-the-art tracking technologies [45]. Thirdly, PSU-related outcome measures are based on self-reporting and brain changes in structure and function between baseline and postintervention were not measured in this study, so potential neurological mechanisms of the beneficial effects of exercise interventions on PSU and the related mental health outcomes remain unclear. There is substantial evidence that many mental illnesses including behavioral addictions are associated with the dopamine-involved brain reward process [46, 47] and that dopamine is also involved in the rewarding aspects of exercise and ultimately the motivation to exercise [48]. The effects of chronic exercise on the dopaminergic system have been extensively verified in animal models at a genetic level [49, 50]. In the context of this research, the larger improvements of PSU and the mental health outcomes introduced by group-based basketball intervention as compared to Baduanjin intervention might be explained as that college students got more dopamine rewards from basketball play which is a more intense, competitive, and game-like than Baduanjin. Future studies should pay attention to changes in dopaminergic functioning introduced by exercise interventions among people with PSU. Fourthly, future studies may include the optimal exercise type or “dose” required for reducing PSU with researches of more exercise interventions in depth. Lastly, it would be of a great value to put more focus on exercise preference patterns among people with PSU for better exercise promotion among them to lower their PSU levels more effectively.

## 5. Conclusions

In conclusion, our interventions demonstrate that both Baduanjin and basketball exercises can effectively reduce PSU of college students and improve their mental wellness with regard to reducing anxiety, loneliness, inadequacy, and stress. Furthermore, such positive effects on all these indices introduced by the two exercises can be sustained for two months after intervention except for the perceived stress index. Future studies might benefit from comparing exercise interventions on this domain with more confounders considered, longer follow-up time designed, and more objective indices measured and from tailoring treatment programs to address the needs of different populations.

## Figures and Tables

**Table 1:** Summaries of baseline variables in the three groups (*N* = 96).

		Baduanjin	Basketball	Control
Age		19.21 ± 1.02	18.95 ± 0.89	19.71 ± 1.77

Gender	Male	24	23	24
	Female	7	8	10

Grade	Freshmen	29	30	28
	Sophomore	2	1	5
	Junior	0	0	1

Major^*∗*^	Science	24	22	34
	Social science	7	9	0

Family	Single child	22	16	22
	Sibling	9	15	12

Class role	Leader	10	4	10
	Nonleader	21	27	24

Dating	Yes	6	5	11
	No	25	26	23

Monthly mobile phone fee (Yuan)	<20	1	2	5
	20–50	12	17	14
	51–100	6	6	9
	101–150	3	0	1
	150–200	9	6	5

Daily mobile phone use (hours)	3-4	0	5	2
	4–6	1	2	4
	6–8	23	18	19
	8–10	3	5	8
	>10	4	1	1

Main use of mobile phone	Social (chatting)	13	16	16
	Study and work	14	13	16
	Personality	0	2	0
	Entertainment	4	0	1
	Killing time	0	0	1

Main social use of mobile phone	Internet surfing	16	14	15
	Movie and music	12	14	18
	Phone and messaging	1	0	0
	Photograph	2	3	0
	Playing game	0	0	1

Awareness of harm of mobile radiation	Know	26	23	30
	Do not know	5	8	4

*Note*. ^*∗*^A significant baseline difference, which was added to the analysis models as the main effect terms to adjust for their potential confounding effects.

**Table 2:** Mean and standard deviation (Mean ± SD) of the raw data values for MPAI, anxiety, stress, inadequacy, and loneliness at the baseline, week 12, and 2-month follow-up in the Baduanjin, basketball, and control groups.

Variable	Baduanjin			Basketball			Control		
	Baseline	Week 12	2-month follow-up	Baseline	Week 12	2-month follow-up	Baseline	Week 12	2-month follow-up
MPAI	51.13 ± 8.66	42.74 ± 6.87	43.48 ± 5.05	48.42 ± 7.80	38.71 ± 4.60	41.03 ± 3.23	47.82 ± 5.83	46.59 ± 5.03	44.85 ± 3.96
Anxiety	51.29 ± 6.00	45.58 ± 5.12	46.42 ± 4.83	52.84 ± 5.05	41.84 ± 4.43	41.58 ± 4.67	54.03 ± 5.31	52.59 ± 5.04	52.85 ± 4.13
Stress	34.23 ± 5.10	29.52 ± 4.3	30.35 ± 4.65	33.87 ± 5.00	27 ± 4.21	32.03 ± 4.05	34.5 ± 5.82	32.68 ± 5.08	31.94 ± 4.79
Inadequacy	76.87 ± 7.71	108.32 ± 15.12	100.52 ± 21.19	78.68 ± 8.23	117.26 ± 15.03	110.81 ± 12.58	77.41 ± 7.88	90.85 ± 14.76	88.18 ± 11.4
Loneliness	50.1 ± 4.82	40.68 ± 2.82	42.45 ± 3.06	50.55 ± 4.51	38.42 ± 2.91	40.13 ± 3.32	48.79 ± 5.76	47 ± 6.2	47.12 ± 4.71

^*∗*^ *p* < 0.05; ^*∗∗*^*p* < 0.01.

**Table 3:** Parameter estimation of time, group, and their interaction effects in the GEE models.

Effect			MPAI	Anxiety	Loneliness	Inadequacy	Stress
Intercept		Coef.	47.54	52.80	49.15	75.59	33.93
		95% CI	(44.41, 50.67)	(50.24, 55.36)	(46.96, 51.33)	(70.40, 80.79)	(31.14, 36.72)
		*p*	0.00^*∗∗*^	0.00^*∗∗*^	0.00^*∗∗*^	0.00^*∗∗*^	0.00^*∗∗*^

Time effect	Time 2	Coef.	−1.24	−1.44	−1.79	13.44	-1.82
		95% CI	(−2.95, 0.48)	(−4.26, 1.38)	(−4.77, 1.18)	(8.03, 18.86)	(-3.94, 0.29)
		*p*	0.16	0.32	0.24	0.00^*∗∗*^	0.09
	Time 3	Coef.	−2.97	−1.18	−1.68	10.76	-2.56
		95% CI	(−4.75, −1.19)	(−3.41, 1.06)	(−4.27, 0.91)	(5.81, 15.72)	(-4.77, -0.35)
		*p*	0.00^*∗∗*^	0.30	0.20	0.00^*∗∗*^	0.02^*∗*^

Group effect	Baduanjin	Coef.	3.24	−3.02	1.38	-0.95	-0.40
		95% CI	(−0.40, 6.88)	(−5.78, −0.25)	(−1.19, 3.96)	(-4.97, 3.07)	(-3.08, 2.28)
		*p*	0.08	0.03^*∗*^	0.29	0.64	0.77
	Basketball	Coef.	0.51	−1.55	1.86	0.74	-0.79
		95% CI	(−2.98, 4.00)	(−4.14, 1.05)	(−0.66, 4.38)	(-3.18, 4.66)	(-3.56, 1.97)
		*p*	0.77	0.24	0.15	0.71	0.57
	Major	Coef.	0.28	1.23	−.3515483	1.81694	.5701275
		95% CI	(−2.17, 2.74)	(−0.62, 3.08)	(−1.41, 0.71)	(-2.67, 6.30)	(-1.44, 2.58)
		*p*	0.82	0.19	0.51	0.43	0.58

Interaction effect	Time 2^*∗*^ Baduanjin	Coef.	−7.15	−4.27	−7.63	18.01	-2.89
		95% CI	(−10.48, −3.82)	(−8.23, −0.31)	(−11.29, −3.96)	(9.46, 26.56)	(-5.61, -0.16)
		*p*	0.00^*∗∗*^	0.04^*∗*^	0.00^*∗∗*^	0.00^*∗∗*^	0.04^*∗*^
	Time 2^*∗*^ basketball	Coef.	−8.47	−9.56	−10.33	25.14	-5.05
		95% CI	(−12.34, −4.61)	(−12.87, −6.25)	(−13.79, −6.88)	(17.43, 32.85)	(-7.52, -2.57)
		*p*	0.00^*∗∗*^	0.00^*∗∗*^	0.00^*∗∗*^	0.00^*∗∗*^	0.00^*∗∗*^

Time 3^*∗*^ Baduanjin	Coef.	−4.67	−3.69	−5.97	12.88	-1.31	
		95% CI	(−8.23, −1.12)	(−6.92, −0.46)	(−9.17, −2.76)	(3.73, 22.03)	(-3.80, 1.18)
		*p*	0.01^*∗*^	0.03^*∗*^	0.00^*∗∗*^	0.01^*∗∗*^	0.30
	Time 3^*∗*^ basketball	Coef.	−4.42	−10.08	−8.74	21.36	0.72
		95% CI	(−7.86, −0.98)	(−13.02, −7.14)	(−12.05, −5.44)	(13.64, 29.09)	(-1.73, 3.17)
		*p*	0.01^*∗*^	0.00^*∗∗*^	0.00^*∗∗*^	0.00^*∗∗*^	0.56

^*∗*^ *p* < 0.05; ^*∗∗*^*p* < 0.01; Time 1: the postintervention time (week 12); Time 2: the 2-month follow-up time after the postintervention time.

## Data Availability

The data used in the study are available upon request to the corresponding author.
